# Venous and arterial thrombosis in patients receiving immune checkpoint inhibitors

**DOI:** 10.1371/journal.pone.0321112

**Published:** 2025-04-01

**Authors:** Anouk van Lent, Rebeca Puscasu, Karin A. H. Kaasjager, Saskia Haitjema, Britt B. M. Suelmann, Marianne C. Verhaar, Meriem Khairoun, Gurbey Ocak

**Affiliations:** 1 Department of Internal Medicine, Sint Antonius Hospital, Nieuwegein, The Netherlands; 2 Department of Nephrology and Hypertension, University Medical Center Utrecht, Utrecht, The Netherlands; 3 Department of Internal Medicine and Dermatology, University Medical Center Utrecht, Utrecht, The Netherlands; 4 Central Diagnostic Laboratory, University Medical Center Utrecht, Utrecht, The Netherlands; 5 Department of Medical Oncology, University Medical Center Utrecht, Utrecht, The Netherlands; BSMMU: Bangabandhu Sheikh Mujib Medical University, BANGLADESH

## Abstract

**Background:**

Immune checkpoint inhibitors (ICPi) have been associated with a prothrombotic and pro-atherogenic tendency which could lead to an increased risk of thrombosis. The aim of this study was to investigate the incidence of venous and arterial thrombosis (myocardial infarction or ischemic stroke) in patients who used ICPi as compared with the general population. Furthermore, we investigated the association between the occurrence of venous or arterial thrombosis and mortality.

**Methods:**

Patients receiving immune checkpoint inhibitors ICPi between January 1, 2013, and May 31, 2020, at the University Medical Center Utrecht, the Netherlands, were included in this study. Indirect standardization was used to compare the incidence rates of venous and arterial thrombosis in patients who used ICPi to the age- and sex weighted incidence rates in the general population. Time-dependent Cox proportional hazard regression model was used to calculate Hazard ratios (HRs) with 95% CIs to investigate the association between the occurrence of a venous or arterial event after start of an ICPi and mortality.

**Results:**

The age- and sex weighted incidence rates in 663 patients who used ICPi as compared to the general population was 22.7-fold (95% CI 16.6–31.0) increased for venous thrombosis, 3.0-fold (95% CI 1.2–7.1) increased for myocardial infarction, and 3.2-fold (95% CI 1.6–5.7) increased for ischemic stroke. After adjustment, the all-cause mortality risk was 2.3-fold (95% CI 1.5–3.5) increased for patients who were diagnosed with venous thrombosis during follow-up and 2.2-fold (95% CI 1.1–4.1) increased for patients who were diagnosed with arterial thrombosis during follow-up as compared with patients without venous or arterial thrombosis during follow-up.

**Conclusion:**

Patients receiving ICPi have elevated risks of venous thrombosis and arterial thrombosis. Occurrence of venous thrombosis or arterial thrombosis during treatment with ICPi is associated with an increased mortality risk.

## Introduction

In the past decade, immune checkpoint inhibitors (ICPi) have been increasingly administrated as innovative treatment of specific types of cancers, such as Hodgkin lymphoma, renal cell carcinoma, urothelial cancer, melanoma, cervix carcinoma and non-small cell lung cancer [1]. ICPi are humanized monoclonal antibodies that block immune inhibitor pathways including programmed death receptor 1 (PD-1) or its ligand (PD-L1) and cytotoxic T-cell lymphocyte-associated protein 4 (CTLA-4) [1,2].

Cancer has been associated with both venous thromboembolisms as arterial thrombotic events, including myocardial infarction and stroke [3]. Active cancer patients constitute 20% of venous thrombosis cases, making it the second most common cause of death in cancer after disease progression [4]. Cancer patients also exhibit a higher prevalence of myocardial infarction [5], with increased mortality risk compared with the general population [6]. Additionally, there is a reported elevated incidence of ischemic stroke in the first year following a cancer diagnosis, with a significant increased risk of fatal stroke [7].

ICPi use in patients with cancer could even further increase the risk of both venous thromboembolisms as arterial thrombotic events. Only limited studies that have investigated the association between ICPi and venous thrombosis (including deep vein thrombosis and pulmonary embolism) or arterial thrombosis (including myocardial infarction and ischemic stroke) in patients who use ICPi [8,9]. ICPi is associated with prothrombotic and pro-atherogenic effects which could lead to increased venous thrombosis and arterial thrombosis risk. ICPi may induce thrombosis by triggering inflammation and coagulation [10]. PD-1 blockage accelerates atherogenesis by increasing pro-inflammatory cells in atherosclerotic plaques [11,12]. Activated T-cells stimulate tissue factor synthesis, potentially promoting hypercoagulability [13,14]. Patients with ICPi-related venous thrombosis show elevated myeloid-derived suppressor cells and inflammatory biomarkers, linking these cells to thrombosis risk [15]. Myeloid-derived suppressor cells may activate platelets and induce endothelium and platelet activation, contributing to thrombosis [15–18].

From a clinical point of view, it is important to know whether venous or arterial thrombosis risks are increased in patients receiving ICPi and whether the development of venous or arterial thrombosis during follow-up is associated with an increased mortality risk. In addition, identification of risk factors could be of help in preventing venous or arterial thrombosis in ICPIs users.

The aim of this study was to investigate the incidence of venous and arterial thrombosis in patients receiving ICPi as compared with the general population. Furthermore, risk factors of venous and arterial thrombosis were investigated. In addition, we investigated the association between venous or arterial thrombosis in patients receiving ICPi and mortality.

## Methods

### Study population

An observational cohort study of patients aged ≥ 18 years who received ICPi at the University Medical Center Utrecht between January 1, 2013, and May 31, 2020 was performed. Patients who received ipilimumab, pembrolizumab, nivolumab, atezolizumab, durvalumab, or tremelimumab (on regular treatment of study treatment base) were included. We excluded patients with missing follow-up data regarding thrombotic events and patients who objected to the use of personal data for research.

This study was performed in accordance with the Declaration of Helsinki. Since the patients were not subjected to interventions and there were no rules of conduct imposed upon them, institutional review board approval was not required. This was determined by the Medical Research Ethics Committee Utrecht. All data were pseudonymized before using it for the analysis. Follow-up started at first ICPi administration and ended with either the occurrence of a thrombotic event, death or the end of follow-up (July 31, 2020).

### Data collection

The Utrecht Patient Oriented Database (UPOD) was used to identify patients receiving ICPi. UPOD is an infrastructure of relational databases based on the hospital-wide electronic patient record systems, comprising characteristics of all patients treated at the University Medical Center Utrecht since 2004 [19]. The patient’s sex, age, body mass index (BMI), comorbid conditions, malignancy type, and date of death were collected through UPOD. Data on smoking behavior, hypertension and medication use were collected manually from the electronic patient’s record. If patients were using antihypertensive medication (Anatomical Therapeutic Chemical Classification Systemcodes C02, C03, C04, C05, C07, C08 and C09) at baseline, then they were defined as having hypertension. Anticoagulant medication was defined as the use of direct oral anticoagulants or vitamin K antagonists. Treatment with chemotherapy during the six months prior to first ICPi administration was recorded. In order to quantify the extent of comorbidities, the Charlson Comorbidity Index was used [20].

### Outcomes

The outcome of the study was the occurrence of a venous thrombosis (including deep venous thrombosis or pulmonary embolism), arterial thrombosis (including myocardial infarction or ischemic stroke) or death. Medical records were searched for the occurrence of an event. According to current diagnostic guidelines symptomatic deep venous thrombosis or pulmonary embolism was considered confirmed when it was diagnosed by compression ultrasound of the leg or by spiral computed tomography, respectively. Myocardial infarction was determined by typical symptoms and electrocardiogram abnormalities, elevated levels of cardiac enzymes, or coronary angiography. Computed tomography or magnetic resonance imaging (MRI) was necessary to diagnose an ischemic stroke.

### Statistical analysis

Continuous variables are presented with means and standard deviation, while categorical variables are presented with counts and corresponding percentages. The follow up time for venous thrombosis was calculated as the time between the start of the study and the end of the study (July 31, 2020) or death or the first episode of deep vein thrombosis or pulmonary embolism during the duration of the study, whichever occurred first. The follow up time for arterial thrombosis was calculated as the time between the start of the study and the end of the study (July 31,2020) or death or the first episode of an arterial event (myocardial infarction or ischemic stroke) during the duration of the study.

Incidence rates for venous thrombosis, myocardial infarction and ischemic stroke were calculated by dividing the number of patients with an event by the total observation time at risk. Indirect standardisation was used to compare the incidence rates of venous thrombosis, myocardial infarction or ischemic stroke to the age- and sex weighted incidence rates in the general population obtained from Danish nationwide cohorts for venous thrombosis [21], for myocardial infarction [22] and for ischemic stroke [23]. We did not have data on incidence rates of venous thrombosis, myocardial infarction and ischemic stroke for different age and sex categories in the Netherlands. Therefore, we used Danish data, since the Danish population resembles the Dutch population in cardiovascular incidence [24].

To investigate the association between risk factors of venous or arterial thrombosis, crude and adjusted hazard ratio and 95% confidence intervals were calculated using Cox proportional hazard regression analyses. The hazard ratios were adjusted for age, sex, BMI, hypertension, smoking, history of deep venous thrombosis, pulmonary embolism, myocardial infarction or ischemic stroke, malignancy type, Charlson Comorbidity index, prior chemotherapy, checkpoint inhibitor type and anticoagulant use. When calculating the hazard ratio of venous thrombosis, we ignored the occurrence of arterial thrombosis and vice versa.

To evaluate the association between the occurrence of a thrombotic event and the subsequent mortality, a time-dependent Cox proportional hazard regression model was used. The hazard ratios were adjusted for age, sex, BMI, hypertension, smoking, history of thrombotic events, malignancy type, Charlson Comorbidity index, prior chemotherapy, checkpoint inhibitor type and anticoagulant use. All analyses were formed using IBM SPSS Statistics version 26.0.

### Results

Of the 678 patients who received ICPi between January 1, 2013, and May 31, 2020, one patient had previously objected to use of personal data for research. Follow-up data regarding thrombotic events were missing in fourteen patients. After the exclusion of these patients, the cohort consisted of 663 patients.

Table 1 shows the baseline characteristics of 663 patients. The mean age was 62 years and 249 (38%) of the patients were female. Of the 663 patients, 77 (12%) patients had a history of venous thrombosis, and 64 (10%) patients had a history of arterial thrombosis (including myocardial infarction and ischemic stroke). The most frequent malignancy type was melanoma (47%), followed by non-small-cell lung carcinoma (24%). Nivolumab (PD-1 inhibitor) (39%) was the most frequently prescribed ICPi. Of the 663 patients, 17 patients used anticoagulant medication at baseline. Of these 17 patients, one patient developed a thrombotic event and 16 patients had no thrombotic event during follow-up.

**Table 1:** Baseline characteristics.

		All patients N = 663		No thrombosis N = 612		Thrombosis N = 51	
Age, mean ( ± SD)		62	± 12.9	62	± 13.0	61	± 11.8
Sex							
	Female (%)	249	(38)	233	(38)	16	(31)
	Male (%)	414	(62)	379	(62)	35	(69)
BMI, mean ( ± SD)		26	±5.7	26	± 5.8	26	± 5.1
Charlson Comorbidity Index, mean ( ± SD)		8	±2.0	8	± 2.0	8	± 2.0
Hypertension (%)		229	(35)	214	(35)	15	(29)
Smoking							
	Never (%)	300	(45)	282	(46)	18	(35)
	Ex-smoker (%)	249	(38)	223	(36)	26	(51)
	Current (%)	114	(17)	107	(18)	7	(14)
History of thrombotic events							
	DVT (%)	31	(5)	29	(5)	2	(4)
	PE (%)	46	(7)	43	(7)	3	(6)
	MI (%)	46	(7)	43	(7)	3	(6)
	Ischemic stroke (%)	18	(3)	17	(3)	1	(2)
Malignancy type							
	Melanoma (%)	314	(47)	296	(48)	18	(35)
	NSCLC (%)	161	(24)	144	(24)	17	(33)
	Gynaecologic cancer (%)	19	(3)	16	(3)	3	(6)
	Urinary tract cancer (%)	79	(12)	72	(12)	7	(14)
	Other (%)	90	(14)	84	(14)	6	(12)
Prior chemotherapy		191	(29)	176	(29)	15	(29)
Checkpoint inhibitor type							
	Nivolumab (%)	198	(30)	184	(30)	14	(28)
	Ipilimumab (%)	45	(7)	42	(7)	3	(6)
	Pembrolizumab (%)	232	(35)	214	(35)	18	(35)
	Combined (%)	129	(20)	120	(20)	9	(18)
	Other (%)	59	(9)	52	(9)	7	(14)
Anticoagulant use		17	(3)	16	(3)	1	(2)

### Venous and arterial thrombosis incidences

The occurrence of thrombotic events after the start of treatment with ICPi is shown in Table 2. During follow-up, 39 of the patients had a venous thrombotic event, of whom 11 were diagnosed with a deep venous thrombosis, 24 with pulmonary embolism and 4 with both. Of the 663 patients, 14 patients had an arterial thrombotic event, of whom 4 were diagnosed with myocardial infarction, 9 with ischemic stroke and 1 with both.

**Table 2:** Thrombotic events after start of ICPi use.

Patients with thrombotic event after ICPi		N = 51	(7.7% of all patients)
Venous thrombotic event		39	(5.9)
	DVT, only (%)	11	(1.7)
	PE, only (%)	24	(3.6)
	Both DVT and PE (%)	4	(0.6)
Arterial thrombotic event		14	(2.1)
	MI, only (%)	4	(0.6)
	Ischemic stroke (%)	9	(1.4)
	Both MI and ischemic stroke (%)	1	(0.2)

Deep venous thrombosis (DVT), pulmonary embolism (PE), myocardial infarction (MI).

**Fig 1:**
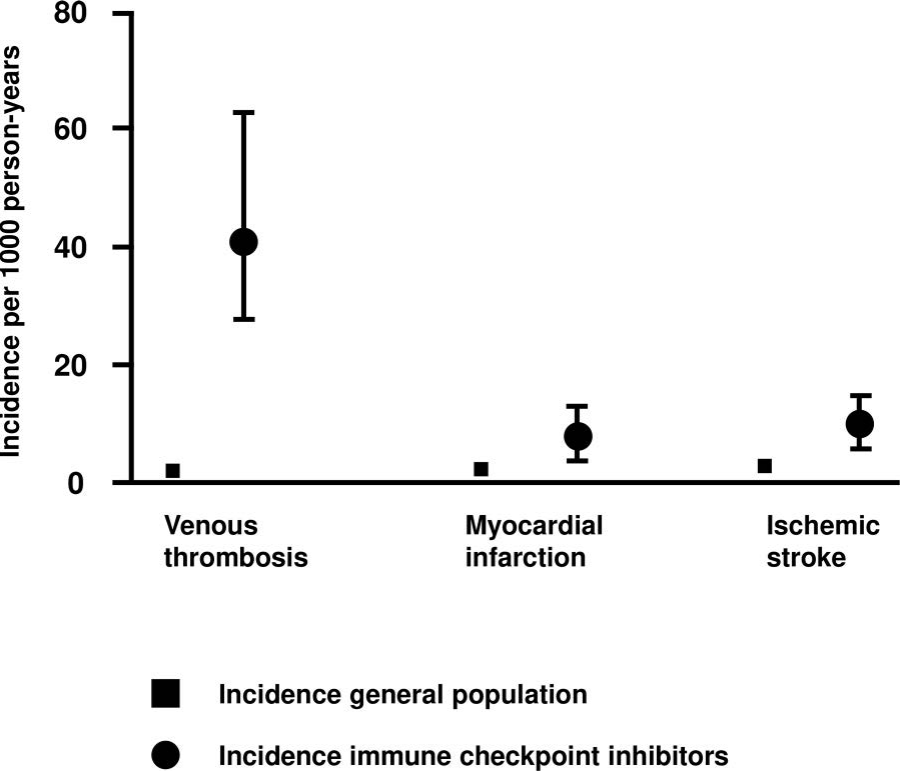
Incidence rates for venous and arterial thrombosis in patient who use immune checkpoint inhibitors as compared to the age- and sex- weighted incidence rates in the general population.

Fig 1 shows the incidence rates per 1000 person-years for venous thrombosis (including pulmonary embolism and deep venous thrombosis), myocardial infarction and ischemic stroke in patients who use ICPi as compared to the age- and sex- weighted incidence rates in the general population. The incidence rate of venous thrombosis (46.3 per 1000 person-years) was 22.7-fold (95% CI 16.6–31.0) increased as compared with the incidence rate in the general population after adjustment for age and sex. Furthermore, the incidence rate of myocardial infarction (5.9 per 1000 person-year) was 3.0-fold (95% CI 1.2–7.1) increased in patients who use ICPi as compared with the age- and sex- weighted incidence rate in the general population. In addition, the incidence rate of ischemic stroke (11.7 per 1000 person-years) was 3.2-fold (95% CI 1.6–5.7) increased as compared with the general population after adjustment for age and sex.

### Risk factors for venous and arterial thrombosis

Table 3 shows the association between several factors and the occurrence of venous thrombosis. After adjustment, gynecologic malignancy was associated with a 6.7-fold (95% CI 1.4–31.4) increased risk of a venous thrombosis. Non-small-cell lung carcinoma and an increased Charlson Comorbidity score showed increased hazard ratios, but this did not reach statistical significance.

**Table 3:** Risk factors for venous thrombosis.

		Hazard Ratio (95% CI)			
		Crude		Adjusted	
Age^1^	< 65	1	(reference)	1	(reference)
	≥ 65	0.7	(0.3–1.3)	0.6	(0.3–1.2)
Sex^2^	Male	1	(reference)	1	(reference)
	Female	0.9	(0.5–1.7)	0.7	(0.3–1.4)
BMI^3^	< 25	1	(reference)	1	(reference)
	≥ 25	1.0	(0.5–1.8)	1.1	(0.6–2.2)
Hypertension^4^	No	1	(reference)	1	(reference)
	Yes	0.7	(0.4–1.5)	0.7	(0.3–1.4)
Smoking^5^	Never	1	(reference)	1	(reference)
	Ex-smoker	2.1	(1.1–4.2)	1.9	(0.8–4.2)
	Current	0.9	(0.3–2.6)	0.7	(0.2–2.1)
History of venous or arterial thrombotic event^6^	No	1	(reference)	1	(reference)
	Yes	0.7	(0.3–1.9)	0.7	(0.3–1.8)
Malignancy type^7^	Melanoma	1	(reference)	1	(reference)
	NSCLC	2.5	(1.1–5.5)	2.2	(0.8–5.9)
	Gynecologic cancer	6.5	(1.8–23.3)	6.7	(1.4–31.4)
	Other	1.1	(0.4–3.3)	1.1	(0.3–4.3)
Charlson Comorbidity Index^8^	0–3	1	(reference)	1	(reference)
	4–7	2.2	(0.3–16.8)	1.8	(0.2–19.3)
	≥ 9	2.0	(0.3–14.6)	2.3	(0.2–27.6)
Prior chemotherapy^9^	No	1	(reference)	1	(reference)
	Yes	1.1	(0.5–2.3)	0.9	(0.4–2.0)
Checkpoint inhibitor type^10^	Nivolumab	1	(reference)	1	(reference)
	Ipilimumab	0.4	(0.1–1.8)	0.4	(0.1–2.2)
	Pembrolizumab	1.1	(0.5–2.5)	1.0	(0.4–2.5)
	Combined	1.5	(0.6–3.9)	1.3	(0.4–3.7)
	Other	1.8	(0.6–5.2)	0.9	(0.3–2.7)

^1^Adjusted for sex, BMI, hypertension, smoking, history of thrombotic events, malignancy type, Charlson Comorbidity index, prior chemotherapy, checkpoint inhibitor type, and anticoagulant use.

^2^Adjusted for age, BMI, hypertension, smoking, history of thrombotic events, malignancy type, Charlson Comorbidity index, prior chemotherapy, checkpoint inhibitor type, and anticoagulant use.

^3^Adjusted for sex, age, hypertension, smoking, history of thrombotic events, malignancy type, Charlson Comorbidity index, prior chemotherapy, checkpoint inhibitor type, and anticoagulant use.

^4^Adjusted for sex, age, BMI, smoking, history of thrombotic events, malignancy type, Charlson Comorbidity index, prior chemotherapy, checkpoint inhibitor type, and anticoagulant use.

^5^Adjusted for sex, age, BMI, hypertension, history of thrombotic events, malignancy type, Charlson Comorbidity index, prior chemotherapy, checkpoint inhibitor type, and anticoagulant use.

^6^Adjusted for sex, age, BMI, hypertension, smoking, malignancy type, Charlson Comorbidity index, prior chemotherapy, checkpoint inhibitor type, and anticoagulant use.

^7^Adjusted for sex, age, BMI, hypertension, smoking, history of thrombotic events, Charlson Comorbidity index, prior chemotherapy, checkpoint inhibitor type, and anticoagulant use.

^8^Adjusted for sex, age, BMI, hypertension, smoking, history of thrombotic events, malignancy type, prior chemotherapy, checkpoint inhibitor type, and anticoagulant use.

^9^Adjusted for sex, age, BMI, hypertension, smoking, history of thrombotic events, malignancy type, Charlson Comorbidity index checkpoint inhibitor type, and anticoagulant use.

^10^Adjusted for sex, age, BMI, hypertension, smoking, history of thrombotic events, malignancy type, Charlson Comorbidity index, prior chemotherapy, and anticoagulant use.

The association between potential risk factors and the occurrence of arterial thrombosis is shown in Table 4. For arterial thrombosis, age, sex, BMI, hypertension, smoking, history of venous or arterial thrombotic event, Charlson Comorbidity Index, prior chemotherapy, and malignancy type were not associated with an increased arterial thrombosis risk. The use of ipilimumab or pembrolizumab showed increased hazard ratios, but this did not reach statistical significance.

**Table 4:** Risk factors for arterial thrombosis.

		Hazard Ratio (95% CI)			
		Crude		Adjusted	
Age^1^	< 65	1	(reference)	1	(reference)
	≥ 65	2.1	(0.7–6.3)	1.6	(0.4–6.8)
Sex^2^	Male	1	(reference)	1	(reference)
	Female	0.7	(0.2–2.1)	0.7	(0.2–2.2)
BMI^3^	< 25	1	(reference)	1	(reference)
	≥ 25	0.7	(0.3–2.1)	0.8	(0.3–2.4)
Hypertension^4^	No	1	(reference)	1	(reference)
	Yes	1.4	(0.5–4.0)	1.3	(0.4–4.0)
Smoking^5^	Never	1	(reference)	1	(reference)
	Ex-smoker	1.0	(0.3–3.4)	0.4	(0.1–1.7)
	Current	1.2	(0.3–4.7)	0.7	(0.2–3.5)
History of thrombotic events^6^	No	1	(reference)	1	(reference)
	Yes	0.74	(0.3–1.9)	0.9	(0.2–3.4)
Malignancy type^7^	Melanoma	1	(reference)	1	(reference)
	NSCLC	4.0	(1.1–14.9)	3.8	(0.7–21.9)
	Gynecologic cancer	–		–	
	Urinary tract cancer	2.8	(0.5–15.9)	2.8	(0.4–19.3)
	Other	1.8	(0.3–10.0)	1.6	(0.1–20.1)
Charlson Comorbidity Index^8^	0–3	1	(reference)	1	(reference)
	4–7	0.3	(0.0–3.0)	0.3	(0.0–7.3)
	≥ 9	0.8	(0.1–6.3)	0.7	(0.0–19.8)
Prior chemotherapy^9^	No	1	(reference)	1	(reference)
	Yes	2.4	(0.8–6.9)	1.7	(0.5–5.8)
Checkpoint inhibitor type^10^	Nivolumab	1	(reference)	1	(reference)
	Ipilimumab	0.6	(0.1–5.6)	1.1	(0.1–17.2)
	Pembrolizumab	1.4	(0.4–4.9)	1.5	(0.3–6.6)
	Combined	0.5	(0.1–4.4)	0.7	(0.1–7.1)
	Other	1.9	(0.3–10.4)	1.4	(0.2–9.2)

^1^Adjusted for sex, BMI, hypertension, smoking, history of thrombotic events, malignancy type, Charlson Comorbidity index, prior chemotherapy, checkpoint inhibitor type, and anticoagulant use.

^2^Adjusted for age, BMI, hypertension, smoking, history of thrombotic events, malignancy type, Charlson Comorbidity index, prior chemotherapy, checkpoint inhibitor type, and anticoagulant use.

^3^Adjusted for sex, age, hypertension, smoking, history of thrombotic events, malignancy type, Charlson Comorbidity index, prior chemotherapy, checkpoint inhibitor type, and anticoagulant use.

^4^Adjusted for sex, age, BMI, smoking, history of thrombotic events, malignancy type, Charlson Comorbidity index, prior chemotherapy, checkpoint inhibitor type, and anticoagulant use.

^5^Adjusted for sex, age, BMI, hypertension, history of thrombotic events, malignancy type, Charlson Comorbidity index, prior chemotherapy, checkpoint inhibitor type, and anticoagulant use.

^6^Adjusted for sex, age, BMI, hypertension, smoking, malignancy type, Charlson Comorbidity index, prior chemotherapy, checkpoint inhibitor type, and anticoagulant use.

^7^Adjusted for sex, age, BMI, hypertension, smoking, history of thrombotic events, Charlson Comorbidity index, prior chemotherapy, checkpoint inhibitor type, and anticoagulant use.

^8^Adjusted for sex, age, BMI, hypertension, smoking, history of thrombotic events, malignancy type, prior chemotherapy, checkpoint inhibitor type, and anticoagulant use.

^9^Adjusted for sex, age, BMI, hypertension, smoking, history of thrombotic events, malignancy type, Charlson Comorbidity index checkpoint inhibitor type, and anticoagulant use.

^10^Adjusted for sex, age, BMI, hypertension, smoking, history of thrombotic events, malignancy type, Charlson Comorbidity index, prior chemotherapy, and anticoagulant use.

### Venous and arterial thrombosis and mortality

Mortality was high in this cohort. During the duration of the study, 362 patients (55%) died corresponding with a mortality rate of 213.4 per 1000 person-years. After the adjustment for age, sex, BMI, hypertension, smoking, history of thrombotic events, malignancy type, Charlson Comorbidity index, prior chemotherapy, checkpoint inhibitor type, and anticoagulant use, the all-cause mortality risk was 2.3-fold (95% CI 1.5–3.5) increased for patients who were diagnosed with venous thrombosis during follow-up. The all-cause mortality risk was 2.2-fold (95% CI 1.1–4.1) increased for patients who were diagnosed with arterial thrombosis during follow-up after adjustment (Table 5).

**Table 5:** Thrombosis and mortality.

	All-cause mortality Crude HRs (95% CI)		All-cause mortality Adjusted HRs (95% CI)	
No venous thrombosis during follow-up	1	(reference)	1	(reference)
Venous thrombosis during follow-up	2.4	(1.6–3.7)	2.3	(1.5–3.5)
No arterial thrombosis during follow-up	1	(reference)	1	(reference)
Arterial thrombosis during follow-up	2.6	(1.4–4.9)	2.2	(1.1–4.1)

HRs, hazard ratios; CI, confidence interval.

## Discussion

In this observational cohort study of 663 patients receiving ICPi, the incidence rate of venous thrombosis was 46.3 per 1000 person-years, the incidence rate of myocardial infarction was 5.9 per 1000 person-year and the incidence rate of ischemic stroke was 11.7 per 1000 person-years. In patients receiving ICPi, the incidence rate of venous thrombosis was 22.7-fold increased, the incidence rate of myocardial infarction was 3.0-fold increased and the incidence rate of ischemic stroke was 3.2-fold increased as compared with the age- and sex- weighted incidence rate in the general population. An association was found between gynecological malignancy and an increased risk of a venous thrombotic event. In addition, patients who were diagnosed with venous thrombosis during follow-up had a 2.3-fold increased mortality risk and patients with arterial thrombosis during follow-up had 2.2-fold increased mortality risk as compared with patients receiving ICPi who did not develop venous or arterial thrombosis.

### Incidence of venous thrombosis

Several studies investigated the occurrence of venous thrombosis events in patients with ICPi use. In one cohort study, the incidence rate for deep venous thrombosis was 0.93 per 1000 person-years (95% CI 0.44–0.56) and for pulmonary embolism 0.50 per 1000 person-years (95% CI 0.44–0.56) [25]. A previous Danish study reported a cumulative incidence of 4.1% (95% CI 2.3%–6.7%) at 6 months for venous thrombosis, with a cumulative incidence of 7.1% (95% CI 4.2%–11.1%) at 12 months [26]. Moreover, an analysis of the prospective APOLLO cohort showed increased venous thrombotic event in patients who used ICPi [27]. We also showed increased incidence of venous thrombosis in our study with longer follow-up or more patients than the previous studies.

### Risk factors of venous thrombosis

Several studies have attempted to identify risk factors for thromboembolic events in patients receiving ICPi. A single-center retrospective cohort study at the Vienna General Hospital, comprising adult patients with histologically confirmed cancer who were treated with ≥  1 dose of an ICPi, showed that patients with gynecological cancer were at higher risk of venous thrombosis [28]. Also, in our study, we found an association between gynecological cancer and an increased risk of venous thrombosis. It could be that patients with gynecological cancer are more immobilized due to surgery or interventions (external radiation therapy, brachytherapy or both). In contrast to our study, several other studies also identified other risk factors of venous thrombosis in patients receiving ICPi. A study showed that a history of thromboembolic disease, metastasis, poor performance status, lung cancer, and melanoma increased venous thrombotic risk [13]. Another cohort study showed that active smoking and age < 65 years were associated with more venous thrombotic events after 12 months of ICPi initiation [29]. Furthermore, a cohort study in patients who received ICPi, found that history of hypertension and history of venous thromboembolism predicted a higher risk of venous thromboembolism, while a history of melanoma predicted a lower risk of venous thromboembolism [30]. In contrast to the above studies [13,28–30], our investigation encompasses a broader spectrum of cancer types, which could be a reason for the discrepant findings.

### Incidence of arterial thrombosis

Limited studies have investigated the risk of arterial thrombosis and ICPi use, which showed increased risks of arterial thrombosis. In a case crossover study, among the 2842 patients who were treated with an ICPi, the incidence rate for a cardiovascular event was 6.55 per 100 person-years compared to 1.37 per 100 person-years for patients who did not use ICPi. In this study, ICPi treatment was associated with a 3-fold higher risk for atherosclerotic (myocardial infarction, coronary revascularization, ischemic stroke) cardiovascular events compared with cancer patients who did not have ICPi after adjustment for age [2]. In another cohort study with 672 patients, the cumulative incidence of arterial thrombosis was 1.6% for patients with a melanoma, 1.4% for patients with a non-small cell lung cancer, 1.7% for patients with a renal cell carcinoma, 2.5% for patients with a nivolumab and 1.5% for patients with a pembrolizumab [28]. In another cohort study, the incidence of arterial myocardial infarction appeared higher than in our study, which could be due to a difference in patient population and characteristics [31].

### Risk factors of arterial thrombosis

There are limited studies that have investigated the risk factors for arterial thrombotic events in patients receiving ICPi. A retrospective study identified non-small cell lung cancer, history of acute vascular events and dyslipidemia as risk factors for atherosclerotic vascular events during treatment with ICPi [32]. A study from the United States identified malignant melanoma and ICPi anti-CTLA-4 monotherapy as risk factors for an ischemic stroke, while patients with neck and head cancers had lower risk of ischemic stroke [33]. In another retrospective cohort study, no risk factors were associated with an increased risk of arterial thromboembolism in patients who were treated with ICPi [28]. This is in accordance with our study in which no risk factors for arterial thrombosis were found.

### Mortality

Limited studies investigated the association between ICPi use, thrombotic events and mortality. In a retrospective cohort study, the all-cause mortality risk was increased for patients who were diagnosed with arterial thrombosis during follow-up. In this study of 228 patients who received ICPi, the overall survival among patients with thromboembolism was worse compared with those without thromboembolism (50.8% versus 71.3%) [34]. In another study, the occurrence of venous thrombosis was associated with a shorter overall survival. After the occurrence of a venous thrombotic event, the overall survival decreased from 25.5 months to 11.6 months [28]. The occurrence of an arterial thrombotic event was not associated with a higher risk of mortality in this study. A Spanish study reported a decreased median overall survival in the group with patients with lung cancer or melanoma who were diagnosed with a venous or arterial thrombotic event [35].

There could be several reasons for the increased mortality rates after venous or arterial thrombosis in ICPi users. First, venous and arterial thrombosis are important causes of cardiovascular mortality in the general population and could also lead to an increased fatality rate in ICPi users. Second, the development of venous and arterial thrombosis in patients with ICPi use could be a marker of disease severity, which could be associated with an increased mortality risk. Finally, it could be that treatment of venous or arterial thrombosis in patients with ICPi use is less effective than in the general population due to the prothrombotic and pro-atherogenic effects of ICPi leading to higher fatality rates than in the general population.

### Clinical relevance

Our observations could have several clinical implications. Firstly, clinicians should be aware that the risks of venous thrombosis, myocardial infarction and ischemic stroke are increased in patients receiving ICPi as compared with the general population. Secondly, the American Society of Clinical Oncology Clinical Practice Guideline on Venous Thromboembolism Prophylaxis and Treatment in Patients With Cancer recommends thromboprophylaxis in high-risk ambulatory patients, including patients with multiple myeloma receiving thalidomide- or lenalidomide-based regimens with chemotherapy or dexamethasone [36]. It could be that (subgroups of) patients receiving ICPi may also benefit from thromboprophylaxis in an ambulatory setting. Future studies should investigate the benefits and safety of thromboprophylaxis in high-risk subgroups of patients receiving ICPi. Future studies should investigate the benefits of these preventive measures in patients receiving ICPi.

## Strengths and limitations

The major strength of this study was the availability of data on confirmed thrombotic events and potential risk factors in a large cohort of patients who used ICPi. Nevertheless, our study has also potential limitations that should be addressed. Firstly, confidence intervals around the hazard ratios were wide when investigating risk factors of venous and arterial thrombosis, indicating a limited power for detecting underlying risk factors for venous and arterial thrombosis. Secondly, the association of arterial or venous thrombosis and mortality could be not causal, as unmeasured confounding is possible. Thirdly, in an observational study, it is difficult to determine the exact interplay and added value of the underlying malignancy and immunotherapy in the thrombosis risk. However, a randomized trial powered on thrombotic events to investigate this point would not be ethical given the positive effects of immunotherapy on survival. Furthermore, from a clinical point of view, the absolute risks of thrombotic events in patients with ICPi use are more important for treatment decisions, such as for the indication of thromboprophylaxis, than the exact risks associated with malignancy alone and added risk of immunotherapy. There could be subgroups of patients who use immunotherapy, such as patients with a gynecologic malignancy, that may benefit from antithrombotic drugs. Future trials and studies are necessary to investigate whether antithrombotic drugs offer benefit in preventing thrombus formation or improve survival. Although this is a single center study, we do not think this has led to selection bias, since immunotherapy is almost exclusively offered in one center in the study region. Fourthly, we had limited power to compare differences or combinations of ICPi in the risk of thrombotic events. Fifthly, we had no information about the causes of mortality. Therefore, we were not able to investigate differences in cardiovascular and non-cardiovascular mortality. Consequently, the impact of these factors on the increased risk of thrombotic events or mortality remains unknown. Another limitation of the study was that there was no information about the ECOG score. Finally, data on response status following immunotherapy treatment was missing. Therefore, we could not investigate the association between the development of thrombotic events and response on immunotherapy.

## Conclusion

In conclusion, patients receiving immune checkpoint inhibitors have increased risks of venous thrombosis and arterial thrombosis compared to the general population and occurrence of venous thrombosis or arterial thrombosis after start of immune checkpoint inhibitors is associated with an increased mortality risk.
